# Somatostatin administration following pancreatoduodenectomy: a case-matched comparison according to surgical technique, body mass index, American Society of Anesthesiologists’ score and Fistula Risk Score

**DOI:** 10.1007/s00595-020-02189-y

**Published:** 2020-12-03

**Authors:** Niccolò Furbetta, Desirée Gianardi, Simone Guadagni, Gregorio Di Franco, Matteo Palmeri, Matteo Bianchini, Kevin Pisani, Giulio Di Candio, Luca Morelli

**Affiliations:** 1 General Surgery Unit, Department of Translational Research and New Technologies in Medicine and Surgery, University of Pisa, Via Paradisa 2, 56125 Pisa, Italy; 2 EndoCAS (Center for Computer Assisted Surgery), University of Pisa, Pisa, Italy

**Keywords:** Somatostatin analogues, Pancreatoduodenectomy, Somatostatin prophylaxis, POPF

## Abstract

**Purpose:**

This study evaluated the controversial role of somatostatin after pancreatoduodenectomy (PD), stratifying patients for the main risk factors using the most recent postoperative pancreatic fistula (POPF) classification and including only patients who had undergone PD with the same technique of pancreatojejunostomy.

**Methods:**

Between November 2010 and February 2020, 218 PD procedures were carried out via personal modified pancreatojejunostomy (mPJ-PD). Somatostatin was routinely administered between 2010 and 2016, while from 2017, 97 mPJ-PD procedures without somatostatin (WS) were performed. The WS group was retrospectively compared with a control (C) group obtained with one-to-one case–control matching according to the body mass index, American Society of Anesthesiologists’ score, and Fistula Risk Score (FRS).

**Results:**

A total of 144 patients (72 WS group versus 72 C group) were compared. In the WS group. 6 patients (8.3%) developed clinically relevant POPF, compared with 8 patients (11.1%) in the C group (*p* = 0.656). In addition, on analyzing the subgroup of high-risk patients according to the FRS, we did not note any significant differences in POPF occurrence. Furthermore, no marked differences in the morbidity or mortality were found. Digestive bleeding and diabetes onset rates were higher in the WS group than in the control group, but not significantly so.

**Conclusions:**

The results of the present study confirm no benefit with the routine administration of somatostatin after PD to prevent POPF, even in high-risk patients. However, a possible role in the prevention of postoperative digestive bleeding and diabetes was observed.

## Introduction

In recent decades, technical evolution and perioperative management improvements have drastically reduced mortality following pancreatoduodenectomy (PD) in high-volume centers [1]. However, morbidity rates remain as high as 28–58%, with post-operative pancreatic fistula (POPF) still the main cause of this high morbidity rate, showing a reported incidence of 2–42.5% [2]. The differences in the incidence of POPF may be due to the varying definitions reported in published studies. Indeed, in 2005, the International Study Group on Pancreatic Fistulas (ISGPF) released a universal classification of fistulae, with Grade B and C fistulae considered clinically relevant [3]. In 2016, the ISGPF reconvened as the International Study Group on Pancreatic Surgery (ISGPS) to update and revise the POPF definition and grading system [4], and the criteria for its diagnosis underwent a radical change.

Significant efforts have been put toward identifying risk factors able to predict POPF formation over the years. The most well-validated predictive model is the Fistula Risk Score (FRS), which stratifies the patient risk according to the pancreatic duct diameter, gland texture, intraoperative blood loss, and pathology [5–7]. Furthermore, several studies have demonstrated a correlation between the development of POPF and other patient-related factors, such as high values for the body mass index (BMI) and American Society of Anesthesiologists’ (ASA) score [8].

Since POPF strongly influences both the short- and long-term outcomes following PD [9–12], various strategies have been adopted in an attempt to reduce the rate of this complication, including the use of fibrin sealants, transanastomotic stents, different techniques for fashioning the pancreatojejunostomy, and the administration of somatostatin analogues (SAs) [13]. Klempa et al. [14] first reported that somatostatin reduced the incidence of complications following PD. Since then, numerous trials have investigated the efficacy of the administration of somatostatin and its analogues to inhibit pancreatic exocrine secretions in an attempt to reduce the risk of POPF.

However, several major concerns still afflict the majority of trials available in the literature, such as marked heterogeneity regarding surgeons and institutional experience, the type of pancreatic resection, and reconstruction technique, as well as the POPF definition and classification and, above all, the lack of fistula risk stratification. For these reasons the strength of recommendations concerning the use of SAs in patients undergoing pancreatic resections remains low, and a majority of centers no longer use them routinely. Nevertheless, a consensus has not yet been reached, and the current position statement of the ISGPS [15] affirms that the routine use of SA may be relevant in high-risk patients.

The primary aim of this study was to evaluate the role of somatostatin in preventing POPF after PD, including an analysis of the data from a tertiary care center by stratifying the risk of POPF using the most relevant risk factors (the FRS [5–7], BMI, and ASA [8, 16]), adopting the updated 2016 ISGPS criteria for POPF definition and classification [4], and including only patients who had undergone PD with the same standardized technique of pancreatojejunostomy [17]. The secondary aim of the study was to evaluate the outcome of patients undergoing pancreatoduodenectomy with and without somatostatin administration in terms of morbidity and mortality.

## Methods

### Data collection

From October 2008 to February 2020, a total of 601 pancreatic resection procedures were performed at the General Surgery Unit, University of Pisa. Among these, 382 were PD, of which 218 were carried out with a personal modified invaginated pancreatojejunostomy technique (mPJ), introduced in November 2010 [17]. Somatostatin was routinely administered after PD with the mPJ technique (mPJ-PD) between November 2010 and December 2016, while from January 2017, the policy was completely changed, and somatostatin prophylaxis was never administered, not even in high-risk patients. Therefore, from January 2017 to February 2020, 97 mPJ-PD procedures without somatostatin (WS group) were performed. In this cohort, neither somatostatin nor its analogues were administered as prophylaxis, only being given as adjuvant therapy in select patients who developed clinically relevant POPF.

In the present study, the WS group was retrospectively compared with a control (C) group of mPJ-PD in which somatostatin had been routinely administered. The two groups were matched using a one-to-one case–control design, where each patient of the WS group was matched with a comparable patient according to the FRS [5–7], ASA score [8], BMI, and pancreatojejunostomy technique (mPJ). The two matched groups were compared for their intra- and post-operative outcomes, with particular attention paid to POPF. Data were retrieved from the institutional prospectively collected dedicated database. In analyzing the data, the first 20 patients who had undergone mPJ-PD were removed to eliminate bias related to the learning curve of the new modified technique.

The preoperative patient characteristics were recorded, including the age, gender, BMI, nutritional status, ASA score, and comorbidities. The preoperative workup included abdominal ultrasonography, chest radiography, abdomen computed tomography (CT), and/or magnetic resonance imaging findings. Patients who had undergone preoperative CT were excluded from the matching in order to reduce possible selection bias. Perioperative data included the operative findings, operative time, estimated blood loss, need for blood transfusions, pancreatic texture, pancreatic duct size, need and type of vascular resection, and pathological findings. Based on the gland texture, pancreatic duct size, histological diagnosis, and estimated intraoperative blood loss, the FRS was calculated, and patients were stratified into four risk classes: negligible, low, moderate, and high.

Postoperative data included the length of hospital stay and morbidity. Morbidity included intra-abdominal fluid collection, wound infection, POPF, delayed gastric emptying, biliary fistula, bleeding, intestinal obstruction, and pulmonary or urinary tract infections. Postoperative complications were graded using the Clavien–Dindo classification [18]. Re-admission rates within 90 days were also tabulated, as well as mortality within 30 days after surgery.

### Surgical and perioperative management

All procedures were performed with the same mPJ technique [17], which merges the two most popular methods of creating PJ reported in the literature [19–21], combining certain technical aspects of end-to-side duct-to-mucosa and some invagination technique along with personal technical details. Since the introduction of this new PJ technique, the perioperative management of patients undergoing PD at our institution has been standardized with regard to the surgical technique, drain placement and removal policy, timing and content of meals, and evaluation and management of POPF. In particular, anastomotic drains were placed in all cases, and POPF occurrence was assessed with daily measurement of the drainage output volume and amylase content on post-operative days 3 and 5 and, when positive, every three days until drain removal. In cases with multiple drains, the highest concentration of drain amylase was used to ascertain whether or not a biochemical leak had occurred. The drainage tubes were removed on POD 5 in patients judged to have an ISGPS grade of “none” and without any signs of intra-abdominal infection or decreasing amylase concentrations from peripancreatic drains.

An abdominal ultrasound examination was performed as a first-level exam in case of any clinical suspicion of intra-abdominal complications and then such cases are followed up by CT. Intra-abdominal collections caused by POPF were drained with an interventional ultrasound procedure, usually involving the placement of a pigtail catheter in the collection in the first instance, with CT-guided pigtail placement reserved for a failed ultrasound-guided procedure. When prophylactic somatostatin was used, it was initiated intra-operatively at the beginning of the mPJ fashioning at 3 mg/12 h intravenously and continued for 5 days. As prophylaxis for postoperative digestive bleeding and to prevent the possible occurrence of an anastomotic gastric ulcer, a proton pump inhibitor (PPI; Pantoprazole) was administered at 40 mg/day intravenously, shifting to oral administration as soon as a semi-liquid diet was well tolerated.

### Fistula classification

POPF was retrospectively classified according to the updated 2016 ISGPS criteria [4]. An asymptomatic POPF that did not require specific treatment and was characterized by elevated drain amylase values (> 3 × upper limit of normal serum amylase concentration) was considered a biochemical leak. Patients who had biochemical leaks with clinical scenarios, such as prolonged management of a surgical drain exceeding 21 days or percutaneous drain placement for abdominal fluid collection, were considered to have Grade B POPF. Grade C POPF classification, which necessitated a major change in the postoperative management and required aggressive clinical treatment, was characterized by any of the following: reoperation under general anesthesia, POPF-related organ failure, or POPF-related death.

### Statistical analyses

The WS and C groups were selected from our prospectively collected institutional database of pancreatic resection using a one-to-one case-matched methodology with Student’s *t* test, where each patient of the WS group was matched with a patient of the C group using the following criteria: gender, age, BMI, ASA score and Fistula Risk Score. Continuous variables are shown as the mean ± standard deviation and were compared using Student’s *t* test. Variables with a non-normal distribution are expressed as the median (IQ1, IQ3) and were compared using Wilcoxon’s test. The chi-square test (or Fisher’s exact test) was used to define associations between categorical factors and the two groups. *p* < 0.05 was considered statistically significant.

## Results

The baseline characteristics of the entire cohort of patients who had undergone mPJ-PD at our institute are shown in Table 1, excluding those of the first 20 who were removed to eliminate possible bias related to the learning curve of the new modified technique.

**Table 1:** Baseline characteristics of the entire cohort of patients who had undergone mPJ-PD before (2010–2016) and after (2017–2020) the routine administration of somatostatin prophylaxis

	2010–2016 (101 patients)	2017–2020 (97 patients)	*p* value
Male, *n* (%)	53 (52.5)	53 (54.6)	0.760
Age (years), mean ± SD	68.2 ± 3.0	70.3 ± 10.5	0.213
Glucose level (mg/dl), mean ± SD	119.9 ± 43.8	128.0 ± 43.2	0.275
Morbidity			
Diabetes, *n* (%)	18 (17.8)	26 (26.8)	0.129
Cardiopulmonary disease, *n* (%)	24 (23.8)	22 (22.7)	0.857
Nutritional status			
Serum protein (g/dl), mean ± SD	6.58 ± 0.82	6.76 ± 0.86	0.223
Albumin (g/dl), mean ± SD	3.55 ± 0.60	3.52 ± 0.58	0.781
BMI (kg/m^2^), mean ± SD	24.88 ± 3.75	24.64 ± 3.79	0.660
Symptoms			
Jaundice, *n* (%)	50 (49.5)	56 (57.7)	0.246
Abdominal pain, *n* (%)	30 (29.7)	32 (33.0)	0.618
Weight loss, *n* (%)	19 (18.8)	20 (20.6)	0.749
Digestive symptoms, *n* (%)	22 (21.8)	18 (18.6)	0.572
Preoperative RT/CT, *n* (%)	2 (2.0)	7 (7.2)	0.077
Pathological findings			0.330
PDAC, *n* (%)	51 (50.5)	60 (61.9)	
IPMN, *n* (%)	4 (4.0)	1 (1.0)	
Pancreatic NET, *n* (%)	7 (6.9)	2 (2.0)	
Other pancreatic neoplasm, *n* (%)	7 (6.9)	6 (6.2)	
Cholangiocarcinoma, *n* (%)	11 (10.9)	6 (6.2)	
Ampullary neoplasm, *n* (%)	10 (9.9)	14 (14.4)	
Gastroduodenal neoplasm, *n* (%)	9 (8.9)	7 (7.2)	
Others, *n* (%)	2 (2.0)	1 (1.0)	

*PDAC* pancreatic ductal adenocarcinoma, *IPMN* intraductal papillary mucinous neoplasm, *NET* neuroendocrine tumor, *BMI* body mass index, *mPJ-PD *pancreatoduodenectomy via personal modified pancreatojejunostomy

The study sample consisted of 144 patients (72 in the WS group and 72 in the C group). The baseline characteristics of the two matched groups are summarized in Table 2, and the matching criteria are shown in Table 3.

**Table 2:** Baseline characteristics of the two matched groups

	C group	WS group	*p* value
Male, *n* (%)	36 (50)	41 (56.9)	0.404
Age (years), mean ± SD	68.71 ± 13.22	69.16 ± 10.79	0.822
BMI (kg/m^2^), mean ± SD	23.96 ± 3.18	24.51 ± 3.33	0.322
Morbidity			
Hypertension, *n* (%)	36 (50.0)	48 (66.7)	0.043
Diabetes, *n* (%)	13 (18.1)	18 (25.0)	0.311
Cardiovascular disease, *n* (%)	15 (20.8)	12 (16.7)	0.522
Pulmonary disease, *n* (%)	3 (4.2)	0 (0)	0.080
Glucose level (mg/dl), mean ± SD	117.15 ± 46.67	127.29 ± 40.35	0.200
Nutritional status			
Serum protein (g/dl), mean ± SD	6.77 ± 0.88	6.59 ± 0.84	0.244
Albumin (g/dl), mean ± SD	3.53 ± 0.59	3.56 ± 0.61	0.727
Symptoms, *n* (%)	58 (80.6)	59 (81.9)	0.831
Jaundice, *n* (%)	35 (48.6)	40 (55.6)	0.404
Abdominal pain, *n* (%)	20 (27.8)	24 (33.3)	0.469
Weight loss, *n* (%)	15 (20.8)	11 (15.3)	0.386
Digestive symptoms, *n* (%)	16 (22.2)	12 (16.7)	0.400
History of abdominal surgery, *n* (%)	42 (58.3)	44 (61.1)	0.734

*BMI* body mass index

**Table 3:** Matching criteria

	C group	WS group	*p* value
BMI			
Underweight, *n* (%)	2 (2.8)	2 (2.8)	1
Normal, *n* (%)	41 (56.9)	41 (56.9)	
Overweight, *n* (%)	26 (36.1)	26 (36.1)	
Obese, *n* (%)	3 (4.2)	3 (4.2)	
ASA score			
ASA II, *n* (%)	17 (23.6)	17 (23.6)	1
ASA III, *n* (%)	47 (65.3)	47 (65.3)	
ASA IV, *n* (%)	8 (11.1)	8 (11.1)	
Fistula Risk Score			
Negligible, FRS = 0, *n* (%)	6 (8.3)	6 (8.3)	1
Low, FRS = 1–2, *n* (%)	14 (19.4)	14 (19.4)	
Intermediate, FRS = 3–6, *n* (%)	41 (56.9)	41 (56.9)	
High, FRS = 7–10, *n* (%)	11 (15.3)	11 (15.3)	

*BMI* body mass index, *ASA* american society of anesthesiologists’ score, *FRS* fistula risk score

Both groups were classified into ASA score groups as follows: 17 ASA II (23.6%), 47 ASA III (65.3%), and 8 ASA IV (11.1%). No differences were found in terms of the operative time (430.28 ± 75.52 min in C group versus 412.07 ± 72.76 min in WS group, *p* = 0.143), intraoperative complications, or blood loss. Intraoperative data and pathological results are shown in Table 4, as well as the staging of pancreatic ductal adenocarcinomas (PDACs).

**Table 4:** Intraoperative data and pathological results

	C group	WS group	*p* value
Operative time (min), mean ± SD	430.28 ± 75.52	412.07 ± 72.76	0.143
Robot-assisted surgery, *n* (%)	0 (0)	2 (2.8)	0.154
Vascular resection, *n* (%)	6 (8.3)	12 (16.7)	0.131
Soft pancreatic texture, *n* (%)	35 (48.6)	38 (52.8)	0.617
Pancreatic duct size			0.486
≥ 5 mm, *n* (%)	25 (34.7)	16 (22.2)	
4 mm, *n* (%)	15 (20.8)	17 (23.6)	
3 mm, *n* (%)	12 (16.7)	12 (16.7)	
2 mm, *n* (%)	8 (11.1)	13 (18.1)	
≤ 1 mm, *n* (%)	12 (16.7)	14 (19.4)	
Blood loss			0.088
≤ 400 ml, *n* (%)	63 (87.5)	53 (73.6)	
401–700 ml, *n* (%)	9 (12.5)	18 (25)	
≥ 700 ml, *n* (%)	0 (0)	1 (1.4)	
Pathological findings			
PDAC, *n* (%)	34 (47.2)	46 (63.9)	
IPMN, *n* (%)	2 (2.8)	1 (1.4)	
Pancreatic NET, *n* (%)	4 (5.6)	1 (1.4)	
Other pancreatic neoplasm, *n* (%)	4 (5.6)	5 (6.9)	
Cholangiocarcinoma, *n* (%)	9 (12.5)	3 (4.2)	
Ampullary neoplasm, *n* (%)	9 (12.5)	10 (13.9)	
Gastroduodenal neoplasm, *n* (%)	8 (11.1)	6 (8.3)	
Others, *n* (%)	2 (2.8)	0 (0.0)	
PDAC staging			0.006
IA	0 (0.0)	5 (10.9)	
IB	2 (5.9)	4 (8.7)	
IIA	6 (17.6)	1 (2.2)	
IIB	21 (61.8)	19 (41.3)	
III	5 (14.7)	17 (37.0)	

*PDAC* pancreatic ductal adenocarcinoma, *IPMN* intraductal papillary mucinous neoplasm, *NET* neuroendocrine tumor, *SD* standard deviation

Each group of patients was stratified according to the FRS into 4 subgroups: negligible risk (6 patients, 8.3%, 0 FRS points), low risk (14 patients, 19.4%, 1–2 FRS points), intermediate risk (41 patients, 56.9%, 3–6 FRS points), and high risk (11 patients, 15.3%, 7–10 FRS points).

For the primary endpoint of the study, POPF was registered in 16 patients (10 BL and 6 grade B fistulas) of the WS group; whereas in the C group, 15 patients developed POPF (7 BL, 7 Grade B, and 1 Grade C), without no significant difference noted between the two groups (*p* = 0.656). Therefore, the rates of clinically relevant POPF were 8.3% in the WS group (6/72 patients) and 11.1% in the C group (8/72 patients), without significant differences. On analyzing the subgroups of patients with a high FRS risk, we found no significant difference in the POPF occurrence (Table 5).

**Table 5:** Incidence of POPF

	No fistula	BL	Grade B	Grade C	*p* value
C group, *n* (%)	57 (79.2)	7 (9.7)	7 (9.7)	1 (1.4)	0.656
Negligible, FRS = 0, *n* (%)	6 (100)	0 (0)	0 (0)	0 (0)	
Low, FRS = 1–2, *n* (%)	12 (85.7)	1 (7.1)	1 (7.1)	0 (0)	
Intermediate, FRS = 3–6, *n* (%)	31 (75.6)	5 (12.2)	5 (12.2)	0 (0)	
High, FRS = 7–10, *n* (%)	8 (72.7)	1 (9.1)	1 (9.1)	1 (9.1)	
WS group, *n* (%)	56 (77.8)	10 (13.9)	6 (8.3)	0 (0)	
Negligible, FRS = 0, *n* (%)	6 (100)	0 (0)	0 (0)	0 (0)	
Low, FRS = 1–2, *n* (%)	11 (78.6)	2 (14.3)	1 (7.1)	0 (0)	
Intermediate, FRS = 3–6, *n* (%)	29 (70.7)	8 (19.5)	4 (9.8)	0 (0)	
High, FRS = 7–10, *n* (%)	10 (90.9)	0 (0)	1 (9.1)	0 (0)	
Total, *n* (%)	113 (78.5)	17 (11.8)	13 (9)	1 (0.7)	

*BL* biochemical leak, *FRS* fistula risk score

For the secondary endpoint of the study, no marked differences were found in terms of the length of the hospital stay (19.57 days in C group versus 19.75 days in WS group, *p* = 0.924) and postoperative complications. The rate of digestive bleeding was higher in the WS group (7 patients, 9.7%) than in the C group (2 patients, 2.8%) (p = 0.085). In addition, the rate of diabetes onset was higher in the WS group (25%) than in the C group (12.5%), almost reaching the statistical significance. (*p* = 0.055). No marked difference was noted in the 30-day mortality rates, with 2 cases in the WS group (2.8%) and 3 in the C group (4.2%); (*p* = 0.6). Postoperative data are summarized in Table 6.

**Table 6:** Postoperative data

	C group	WS group	*p* value
Hospital stay (days), mean± SD	19.57 10.21	19.75 12.00	0.924
Post-operative blood transfusion, *n* (%)	24 (33.3)	16 (22.2)	0.137
30-day mortality, *n* (%)	2 (2.8)	3 (4.2)	0.649
Overall complications			
Cardiological, *n* (%)	5 (6.9)	8 (11.1)	0.383
Pulmonary, *n* (%)	13 (18.1)	10 (13.9)	0.495
Onset of diabetes, *n* (%)	9 (12.5)	18 (25)	0.055
Digestive bleeding, *n* (%)	2 (2.8)	7 (9.7)	0.085
Clavien Dindo			0.936
I, *n* (%)	11 (15.3)	12 (16.7)	
II, *n* (%)	25 (34.7)	19 (26.4)	
IIIa, *n* (%)	2 (2.8)	2 (2.8)	
IIIb, *n* (%)	4 (5.6)	3 (4.2)	
IV, *n* (%)	1 (1.4)	2 (2.8)	
V, *n* (%)	2 (2.8)	3 (4.2)	
30-day reoperation, *n* (%)	2 (2.8)	2 (2.8)	1

## Discussion

For years, surgeons have explored surgical techniques and medical interventions with the goal of preventing POPF, but none have led to the widespread adoption of a new standard of care.

Somatostatin is an endogenous tetradecapeptide with a wide spectrum of action, as it inhibits pancreatic exocrine, biliary, and small bowel secretions and increases the net absorption of water and electrolytes [22]. The concept of inhibiting exocrine pancreatic secretion to prevent postoperative complications originated in 1979, when Klempa et al. [14] first reported that somatostatin reduced the incidence of complications following PD. As somatostatin has a short half-life of between 1 and 2 min, several analogues have been developed to avoid the need for continuous intravenous infusion. The octapeptide octreotide has a half-life of 120 min, allowing for intermittent subcutaneous dosing schedules [22]. Pasireotide is the most recently derived SA and has garnered significant interest in the prevention of POPF, as it shows a broader binding affinity and a better pharmacokinetic profile than other SAs [23].

While previous trials have demonstrated the efficacy of SA prophylaxis in reducing the incidence of POPF [22–24], there has been no consensus regarding their routine prophylactic use for preventing POPF, and some studies have shown that SAs not only have no effect on the incidence of POPF [25, 26] but also incur significant additional costs [27], thus advocating against routine prophylaxis [27, 28]. Furthermore, different patient- and disease-related factors are implicated in POPF development, and many authors have proposed composite Fistula Risk Scores based on such variables [29, 30]. SA prophylaxis alone may, therefore, not be effective for reducing POPF, resulting in the need for more randomized studies to be conducted.

However, the results of numerous trials available in the literature have varied widely due to several major issues. First, many studies lack fistula risk stratification, as they predate the development of the FRS [5]. Second, there is great heterogeneity with regards to the surgeon and institutional experience, pancreatic pathologies included, kind and dosages of SA administered, type of pancreatic resection (PD versus distal pancreatectomy), reconstruction technique used, and policy concerning drain placement and removal. Third, the definition of POPF has yet to be standardized, and most previous trials predate not only the 2016 ISGPS revised POPF definition and classification [4] but also the initial ISGPF classification [3]; studies therefore have not differentiated between biochemical and clinically relevant POPF. Indeed, a recent meta-analysis by Adiamah et al. [31] of 1615 patients from 12 randomized studies including different types of SA administered concluded that the routine administration of an SA cannot be recommended following PD, as it did not improve outcomes. They included studies published after 2000 and separately analyzed studies published after 2005 to include the ISGPF definitions. However, they acknowledged a possible publication bias, as the analysis included papers by authors who did not report separately on the blinding of outcome assessors (detection bias) and claimed to include only PD studies. The studies of Sarr et al. [32], Allen et al. [33], and Katsourakis et al. [34] all included distal pancreatectomy patients.

The most recent meta-analysis by Li et al. [35] reporting on the relationship between the use of different SAs and the occurrence of POPF stands in stark contrast with the above-mentioned previous meta-analysis, concluding that SA prophylaxis significantly reduces the POPF incidence as well as overall morbidity but not mortality. However, several biases may have again affected the results of this study. First, the most recent ISGPS classification of POPF was reported in 2016 [4], and attempts at a universal definition of POPF were first performed in 2005 [3]. Therefore, each individual randomized control trial (RCT) assessed POPF using different definitions, with pre-2005 RCTs including ‘biochemical leak’ within the POPF definition, and studies from 2005 to 2016 following the first POPF classification system that, however, presents dramatic differences in comparison to the 2016 updated one. Furthermore, although the same grading system (Grades A/B/C) was used, the grades themselves were not interchangeable between the 2005 and 2016 definitions. According to the 2005 definitions [3], Grade A POPFs were called “transient fistulae”, as these POPFs require little change in management and are mostly managed by the slow removal of surgical drains. Grade B POPFs were defined as POPFs that require a change in management but, in contrast to the 2016 definitions [4], did not have a requirement of at least three weeks of prolonged drainage. Grade C POPFs, according to the 2005 definition [3], were those requiring a major change in management with aggressive intervention. The 2016 definition [4] is more specific, with Grade C POPF only applied to POPFs that lead to re-operation, single/multi-organ failure or mortality. Although the above-mentioned authors tried to stratify POPFs into BL and clinically relevant POPF (define as Grade B/C POPF) based on the 2016 ISGPS definitions [4], they encountered difficulty in separating POPFs into distinct groups due to heterogeneity in the definition. Even the two most recent randomized studies available [25, 26], published after 2016, started enrolment in 2014 and used the 2005 ISGPF definitions of POPF; furthermore, both these studies were unique, as El Nakeeb et al. [26] performed pancreaticogastrostomy anastomosis, while You et al. [25] exteriorized the pancreatic stent across the pancreaticojejunostomy anastomosis, declining to draw definitive conclusions. Second, the FRS was proposed by Callery et al. [5] to stratify patients who might require SA prophylaxis by risk, with prophylaxis provided to those with higher scores, as also recommended by the current ISGPS position statement [15]. However, despite the fact that the FRS has been used in the past to evaluate the effectiveness of SA prophylaxis, no RCT has yet used it to stratify patients and evaluate the effectiveness of SA prophylaxis [35], especially in high-risk patients.

For these reasons, we tried to eliminate selection biases by stratifying the risk of POPF using a prospectively validated clinical risk score [5–7] involving the updated 2016 ISGPS criteria [4] for POPF definition and classification and including only patients who had undergone PD with the same pancreatojejunostomy technique (mPJ), which has been demonstrated to be a valid and safe alternative to other methods [17]. Indeed, our previous study describing this technique [17] reported a low total rate of POPF (17%), and the rate of POPF in high-risk patients according to the FRS was 18.8%, supporting the safety and efficacy of mPJ, especially for “difficult” pancreas cases with a high FRS (soft gland texture and small duct). Furthermore, when matching patients for the analysis, we also took into consideration two other patient-related risk factors for POPF: the BMI and ASA score. Several different studies have also included these intrinsic patient features in the multifactorial pathogenesis of POPF, identifying obesity as a significant predictor of POPF [36], so BMI is one of the most consistent and frequently included variables in the various risk scores proposed in the literature. Ellis et al., in a study including 15,033 PDs, confirmed a higher BMI to be a predictor of POPF both in bivariate and multivariate models [37]. Wiltberger et al. reported on the outcomes of 405 patients who underwent PD, finding that the ASA score and BMI were two independent significant predictors of postoperative major complications in their multivariate analysis [16].

The general trend has not been in favor of the routine clinical use of somatostatin or its analogues and our institutional policy of recent years was in line with this trend. However, we believe that this analysis, which aimed to overcome the previously described limitations of the available literature and provided specific information on high-risk pancreas cases, thanks to stratification, successfully added new information to a still-controversial debate. However, due to the reported considerations, this topic is far from being completely resolved.

In our series, even after adjusting for all possible confounding factors, we did not find any statistically significant difference between the two groups in terms of POPF occurrence, confirming the appropriateness of the aforementioned general trend. Furthermore, although the current position statement of the ISGPS suggests that the routine use of SAs may be relevant following pancreatoduodenectomy for high-risk patients only [15], we did not note any significant difference even when considering only the subgroups of patients at a high risk of developing POPF according to the FRS. Thus, our experience ultimately does not support this recommendation if the aim is to prevent POPF, even in high-risk pancreata.

However, other topics of discussion have emerged from the present study. Indeed, while we found no statistically significant difference between the two groups in terms of the mortality and overall morbidity, we did note a difference in the rates of postoperative digestive bleeding, suggesting that somatostatin administration may play a role in this setting. The observed digestive bleeding rates obviously do not refer to bleeding directly related to erosive POPF but rather to minor digestive bleeding that clinically manifested as hematochezia or melena, despite the same PPI therapy being administered to all patients in both groups. Somatostatin has been proven to reduce the portal blood flow or hepatic venous pressure gradient in most experimental studies [38], so SAs are still used for the emergency treatment of bleeding oesophageal varices in cirrhotic patients. SAs are also used to treat gastrointestinal angiodysplasias, as several observational studies and meta-analyses have suggested that SAs can reduce the re-bleeding and transfusion requirements in patients with gastrointestinal angiodysplasias bleeding refractory to or inaccessible by endoscopic therapies [39]. Furthermore, multiple studies have correlated SAs with a reduction in the pancreatic perfusion and gastroduodenal mucosal blood flow [40–43], and this reduction seems to selectively affect the splanchnic circulation without altering the systemic circulation [42]. McMillan et al. [44], in a retrospective study including a risk-adjusted analysis of 1018 patients who had undergone PD, reported that SA administration not only did not lead to any clinical benefit but was associated with an increased rate of POPF, especially in high-risk patients. The authors explained that these results suggested that the change in pancreatic and gastroduodenal perfusion caused by SA resulted in a reduced anastomotic perfusion, thereby promoting ischemia and congestion at the site of the pancreatic anastomosis, limiting or impairing the wound healing, and consequently increasing the risk of POPF. In contrast, we found no notably high rate of POPF in the C group, so we suspect that the regionalized ischemia induced by somatostatin was the reason for the lower rate of postoperative digestive bleeding in the C group, despite no observable effect on POPF development. Even if our results concerning this point are not statistically significant and the digestive bleeding observed was not clinically relevant, further studies should investigate the possible role of somatostatin in patients at a high risk of bleeding rather than in those with a high FRS.

The other interesting result that emerged from this study was the rate of the onset of diabetes, which was higher in the WS group to a nearly significant degree. The involvement of hyperglucagonemia, which is suspected to be even more important than insulin deficiency in the pathogenesis of diabetes [45], might explain this result. Indeed, patients with diabetes are characterized by not only a compromised insulin secretion and action but also paradoxically elevated plasma concentrations of glucagon that fail to decline appropriately or even increase in response to an oral glucose tolerance test (OGTT) [46]. Thus, somatostatin may represent an attractive approach for treating post-pancreatectomy diabetes by reducing glucagon secretion and improving glucose tolerance. Previous reports have shown that both the fasting and postprandial blood glucose levels during OGTT decreased after the administration of somatostatin in patients who had undergone total pancreatectomy [45, 47, 48], suggesting an extrapancreatic origin of postprandial hyperglucagonemia. Indeed, the discovery of extrapancreatic glucagon secretion and the postprandial hyperglucagonemia observed in totally pancreatectomized patients has changed the concept of glucagon to a pancreas-specific hormone and thus proposed the theory of postprandial hyperglucagonemia as a gut-dependent phenomenon [46]. Glucagon is a product of the preproglucagon gene (GCG), which is expressed by both pancreatic α-cells and specific enteroendocrine cells (L cells) of the intestinal mucosa and neurons within the nucleus of the solitary tract. It is, therefore, possible that the proglucagon-containing enteroendocrine L cells may be the origin of the extrapancreatic glucagon secretion. In this regard, the results of the study by Lund et al. [46] raise the interesting possibility of an increased recruitment of GCG-expressing cells in pancreatectomized patients as a response to the removal of the pancreatic α-cells. These considerations are not totally applicable to our study, as our cohort did not undergo total pancreatectomy. Unfortunately, there are no data available at present concerning PD, so further studies will be needed to better understand the possible role of somatostatin in preventing the onset of postoperative diabetes in this setting.

A final aspect to consider concerns the different SAs available and tested in addition to somatostatin, such as octreotide, vapreotide, and pasireotide. These SAs have varying pharmacokinetic and pharmacodynamic profiles. In the present study, we only considered patients treated with somatostatin, as it historically represents the most commonly used SA at our institution as well as the most economically viable one. Both of the most recent meta-analyses mentioned above [31, 35] are limited by their consideration of all SAs indifferently, and thus far, there have been no randomized trials comparing two or more different analogues. Every SA has always been studied individually, with different and controversial results. Pasireotide seems to be the most promising of the currently available SAs, as Allen et al. found in their 2014 study, the only randomized double-blinded trial available in the literature [33]. Those authors found prophylactic pasireotide to be effective for reducing POPF. However, subsequent studies have not unanimously confirmed these encouraging results, so further studies are required.

In conclusion, despite the limitations of this study, mainly due to its retrospective nature and relatively small sample size, we used a rigorous case-match methodology stratifying patient according to the main risk factors: FRS, ASA score, and BMI. In addition, the surgical approach to pancreatic reconstruction was standardized [17], and POPF was strictly defined and classified using the new ISGPS grading [4] to reduce bias. The results of the present study confirm no benefit associated with the routine administration of somatostatin after PD to prevent POPF, not even in high-risk patients, and not even in terms of the overall postoperative morbidity and mortality. However, although not statistically significant, improved outcomes in terms of postoperative digestive bleeding and diabetes onset were observed in patients treated with somatostatin, suggesting once again that the debate on the use of SAs following PD is far from over. Further studies are necessary to validate these results and draw definitive conclusions.
